# Why language matters: insights and challenges in applying a social *determination* of health approach in a North-South collaborative research program

**DOI:** 10.1186/s12992-015-0091-2

**Published:** 2015-02-27

**Authors:** Jerry M Spiegel, Jaime Breilh, Annalee Yassi

**Affiliations:** School of Population and Public Health, Department of Medicine, the University of British Columbia, Rm. 430 – 2206 East Mall, Vancouver, V6T 1Z3 BC Canada; Health Sciences Area, University Andina Simon Bolivar, Quito, Ecuador

**Keywords:** Social determination of health, Social determinants of health, North-South collaboration, Health equity

## Abstract

**Background:**

Focus on “social determinants of health” provides a welcome alternative to the bio-medical illness paradigm. However, the tendency to concentrate on the influence of “risk factors” related to living and working conditions of individuals, rather than to more broadly examine dynamics of the social processes that affect population health, has triggered critical reaction not only from the Global North but especially from voices the Global South where there is a long history of addressing questions of health equity. In this article, we elaborate on how focusing instead on the language of “social determination of health” has prompted us to attempt to apply a more equity-sensitive approaches to research and related policy and praxis.

**Discussion:**

In this debate, we briefly explore the epistemological and historical roots of epidemiological approaches to health and health equity that have emerged in Latin America to consider its relevance to global discourse. In this region marked by pronounced inequity, context-sensitive concepts such as “collective health” and “critical epidemiology” have been prominent, albeit with limited acknowledgement by the Global North. We illustrate our attempts to apply a social determination approach (and the “4 S” elements of bio-Security, Sovereignty, Solidarity and Sustainability) in five projects within our research collaboration linking researchers and knowledge users in Ecuador and Canada, in diverse settings (health of healthcare workers; food systems; antibiotic resistance; vector borne disease [dengue]; and social circus with street youth).

**Conclusions:**

We argue that the language of social determinants lends itself to research that is more reductionist and beckons the development of different skills than would be applied when adopting the language of social determination. We conclude that this language leads to more direct analysis of the systemic factors that drive, promote and reinforce disparities, while at the same time directly considering the emancipatory forces capable of countering negative health impacts. It follows that “reverse innovation” must not only recognize practical solutions being developed in low and middle income countries, but must also build on the strengths of the theoretical-methodological reasoning that has emerged in the South.

## Background

Focus on the “social determinants of health” as a way to “close the [health] gap in a generation” [1] provides a welcome alternative to the bio-medical paradigm’s excessive focus on genetic and clinical factors. However, there remains a strong tendency for public health practitioners and traditional epidemiologists to continue to concentrate on the influence of “risk factors” related to living and working conditions of individuals, rather than to more systematically address the social *dynamics* that affect population health. This persistence has triggered critical reaction from voices not only in the Global North (e.g. [2-4]) but especially in the Global South [5-7] where there has been a long history of examining the social processes impeding healthy living (*buen vivir*) [8-12]. In this article, we discuss how adopting a language that focuses more directly on the social processes underlying health inequities can prompt a different approach to research and related policy and praxis. We see this discursive change in line with Bourdieu’s recognition of the “symbolic power” associated with how science is invoked at any given time as an expression of social and political relations [13]. By focusing on how a language that has been developed more extensively in the Global South can inform empirical research in global health, we seek to extend the “reverse innovation” debate that has been raised in the *Globalization and Health* journal.

Health equity, an expression increasingly embraced in the Global North, has been defined as “the absence of systematic disparities in health or in the major social determinants of health, between social groups who have different levels of underlying social advantage/disadvantage—that is, different positions in a social hierarchy” [14]. In this regard, Solar and Irwin [15], clearly distinguish between what are termed "structural determinants” and “intermediary determinants” in their conceptual framework for action on the social determinants of health, published as a World Health Organization (WHO) discussion paper:

The central role of power in the understanding of social pathways and mechanisms means that tackling the social determinants of health inequities is a political process that engages both the agency of disadvantaged communities and the responsibility of the state. Second, it is important to clarify the conceptual and practical distinction between the social causes of health and the social factors determining the distribution of these causes between more and less advantaged groups. The CSDH [Commission on Social Determinants of Health] framework makes a point of making clear this distinction. On this second point of clarification, conflating the social determinants of health and the social processes that shape these determinants’ unequal distribution can seriously mislead policy. ….. The vocabulary of “structural determinants” and “intermediary determinants” underscores the causal priority of the structural factors (pp.5-6).

Concurring with this overall analysis and especially the caution about “conflating the social determinants of health and the social processes that shape these determinants”, we note that the *language* of “social determinants” (SDH) still tends to invite a targeting of individual, or at best community risk factors as the site of population health interventions without necessarily calling attention to hierarchical dynamics and social processes - and can thus tolerate reductionist framings of causal association. Joining colleagues from the Global North who also call for more comprehensively concentrating on systemic power relations (e.g. [2-4]), researchers from the Global South have been arguing for shifting our attention to social processes (e.g. [16]), and indeed shifting our language from “social determinants” to the processes of the “social determination” of health [SDnH] ([17-20]).

That this orientation has received considerable attention in Latin America has been noted by numerous observers [16,21-24], despite its relative invisibility in the CSDH discussion of historical antecedents to the appreciation of “social determinants”. The emergence in Latin America of a different conceptualization of “health equity” has scarcely taken place in a vacuum. After all, in an increasingly globalized world marked by growing power asymmetries, the tendency in mainstream thinking to uncritically apply conceptual and epistemological framings dominant in “Northern” settings has undervalued understandings that have emerged in the Global South [21,25]. This has been well recognized by the editors of *Globalization and Health* in their explicit call for learning from the South [26]. Crisp [27], noting that “*there is still relatively little reverse flow of ideas and approaches from lower to higher income institutions” (p.2),* echoes the arguments of Barrios Suarez et al. [28] lamenting the typical North to South direction of global health knowledge rather than a truly reciprocal bi-directional flow*.* This Debate article similarly argues that “talking from the South” not only implies focus on *problems* experienced in this locale, but also calls for building on the strengths of the *theoretical-methodological reasoning* that has emerged in the South.

As distinctions in terminology may appear to be subtle, our Discussion below begins by briefly revisiting the theoretical and conceptual underpinnings that drive analysis of health and their respective evolutions in the Global North and the Latin American “Social Medicine”/“Collective Health” traditions. This is accompanied by a brief discussion of the nature of the “health equity/social determinants of health” dominant framing and the parallel work that has been taking place on the “social determination” of health conceptualization, briefly comparing and contrasting the two. Here we point to why language that re-focuses on the social processes (or SDnH) is more likely to lead to insights and actions that contribute to sustainable health equity. Such critical social theory considerations are especially pertinent in South-north global health research collaborations where teams are working together in participatory action research and other participatory empirical studies. As such, our Ecuadorian-Canadian partnership [29] provides an excellent opportunity to contribute to this analysis. In this context we introduce our long-standing (now decade long) international collaborative research program that currently addresses five quite distinct population health problems and settings, (health of healthcare workers; food systems; antibiotic resistance; vector borne disease [dengue]; and social circus with street youth), illustrating our attempts to apply the rich social theory from Latin America into the empirically rich participatory research we are conducting in diverse contexts. We conclude by reflecting on the broad epistemological and empirical challenges as well as opportunities that are presented in pursuing North-south collaborations.

## Discussion

### The paradigm clash in epidemiology

A thorough historical overview of the evolution of the various epistemological tendencies in public health was provided by Waitzkin [30]; and the political dimensions of the current debate analyzed more recently by Birn [4]. Despite the origins of public health being thoroughly rooted in profound appreciations of social and political contexts (Virchow, Engels) as described by Birn for example [4], the insights provided by breakthroughs in biological and medical sciences led by the emergence of germ theory in the late 19th century set the stage for new approaches. Importantly, while the evolution of epidemiological study prompted the development of increasingly sophisticated and powerful statistical and design techniques for measuring associations of exposure and disease, such “success” served to marginalize more theoretically-rich interdisciplinary - let alone transdisciplinary and intercultural - approaches to understanding the determination of disease in populations. Recognizing the challenges that tended to have been relatively ignored by mainstream “*risk factor” epidemiology*, new orientations and scientific paradigms related to *social epidemiology* and population health began to emerge in the Global North in the latter half of the twentieth century, cognizant of the intensifications of complexity-rooted effects and disparities that were being generated by ever intensified social changes (e.g. see the work of Krieger [31,32]).

Meanwhile alternative framings for more explicitly putting social justice and associated action at the core of the scientific endeavour were emerging in precisely the settings where marginalization and disparity were more intense. Specifically, in Latin America over the course of the Twentieth Century, the visible signs of extreme social and political authoritarianism and inequity, as well as the growing unfairness of the world economy, inspired a culture of social critique - and a corresponding academic reform movement related to health research began to be entrenched in major public universities in Latin America. It was no coincidence that this tendency paralleled the innovative orientation to learning prompted by Paolo Freire’s *Pedagogy of the Oppressed* in response to formalistic approaches to learning and in search of social justice [33]. Such circumstances nurtured a profound social awareness among health scientists whose academic or public health roles placed them in direct contact with the devastating effects of poverty. This is the controversial trajectory under which epidemiology developed in Latin America since the late 1970s, transforming from a basic *knowledge formation* built around certain processes to a *discipline* constructed around partially defined objects, to finally becoming a *science* structured around clearly defined objects of study [34]. As various North American scholars noted, however, “two of the most significant developments in health scholarship and practice of our era - the social medicine and critical epidemiology movements in Latin America” [35], were remaining largely unknown in the North. In fact, there has been limited direct interchange between the scholarship in the South and the more counter-hegemonic expressions of epidemiology in the Global North [22]. As such, the timing remains overripe for pursuing active collaborations, such as the partnership in which we are engaged, as discussed below.

In Latin American academic environments, reflection about a new critical health theory has linked three crucial elements that are inherently interrelated: health as an *object*; health as a *methodological concept,* and health as a *field of action* [36]. As Breilh [7] has elaborated, Latin American researchers have insisted that in order to develop a critical epidemiological paradigm, it is necessary to intertwine three complementary transformations: first, re-conceptualizing health as a complex, multidimensional object, submitted to a dialectical process of determination; second, innovation in methodology and techniques of researching health; and, third, a transformation of the practical applications and mobilization of social forces.

In the SDnH conceptualization, the social inequities at the macro level are portrayed explicitly as dynamic interconnected social and socio-natural (metabolic^a^) processes in a dialectical relationship with mezzo elements, directly shaping the *modes* or *ways* of living of communities within the broader context that in turn influence *styles of living* at the micro individual/family scale. In this way, attention is directly drawn not only to systematically consider the health effects associated with “determinants”, but also to consider the processes whereby those who are affected also *respond* to these circumstances, within and across scales. In this sense, those affected are considered as subjects whose *agency* is directly part of the theoretical conceptualization. In contrast, using the language of SDH and conventional epidemiology, interventions can focus on identified risk factors. The agency of those affected by these determinants tends to be relatively secondary in such analyses, and added as a caveat to consider, as per Solar and Irwin’s cautions [15], rather than as central to the conceptual design of the relationships under consideration.

**Figure 1:**
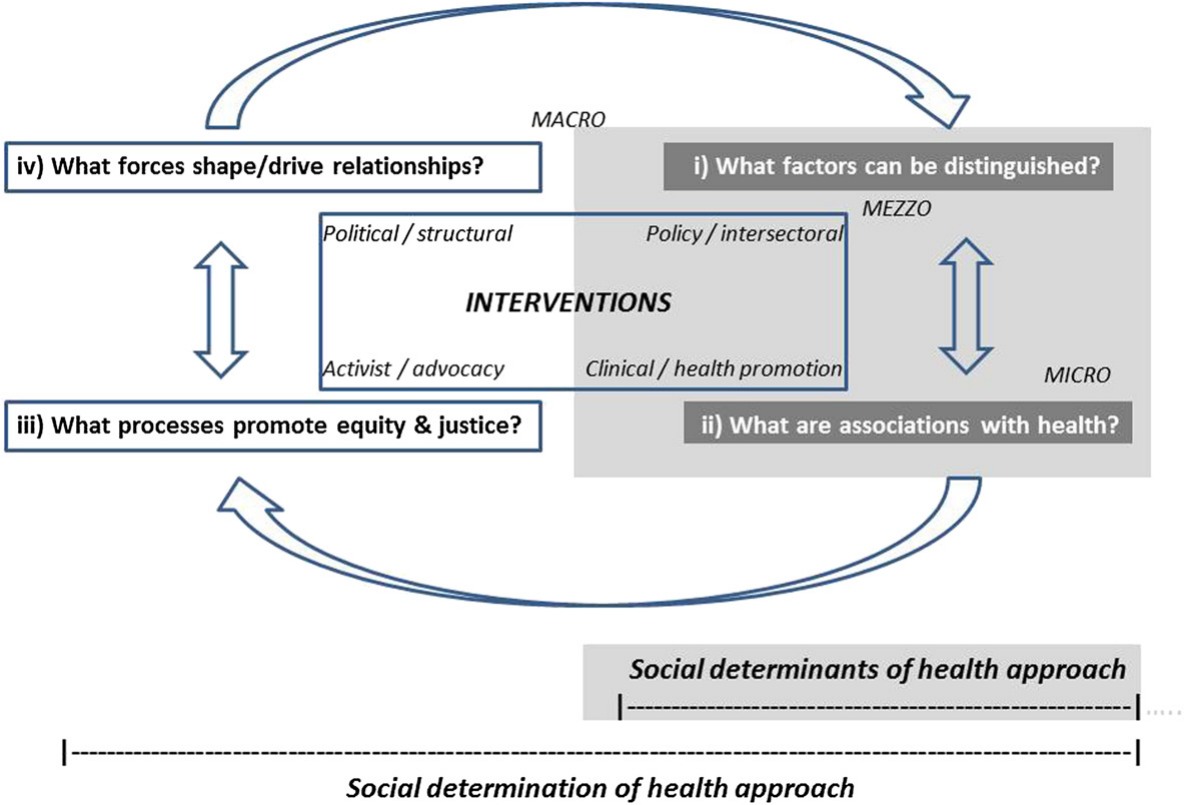
**Framing social determination versus social determinant orientation.**

In the SDH orientation articulated within mainstream epidemiology, the driving questions that target the relationship between social determinants and health equity can be characterized as i) “what factors can be distinguished” as exerting influences on health, and ii) “what are the associations with health”. In common with the many other critics mentioned above, our conceptualization of the social determination approach sees these considerations as necessary but not sufficient to dynamically consider driving influences as well as how affected social actors can engage with the normative position of pursuing social justice and health equity. In other words, as illustrated by Figure 1, our SDnH approach more directly promotes consideration of iii) processes that may be supportive of “emancipation” and health equity (potentially challenging hegemonic status quo social relations); and iv) the forces that drive and influence relationships and determinants themselves, over and above a role in “determining the distribution of these causes between more and less advantaged groups” as per the SDH conceptual framing [15].

Competing perspectives on how influences on health could and should be framed came to a head at meetings held in Rio de Janeiro, Brazil in 2011, when a collection of civil society organizations that were independent and critical of the governmental representatives who were meeting to chart a course for the WHO Commission on Social Determinants of Health, expressed concerns in the following way:

The social determination of health is much more than a collection of fragmented and isolated “determinants” that, from a reductionist viewpoint, [is] associated with classic risk factors and individual lifestyles. We must not allow the concept of social determinants of health to become banal, co-opted or reduced merely to smoking, sedentary behavior and poor nutrition, when what we need is to recognize that behind those symptoms and effects lies a social construction based on the logic of a globalized hegemonic culture whose ultimate goal is the commercialization of life itself [37].

Building on the work of many critics in both the Global North and the Global South, the SDnH approach that we wish to highlight has been primarily developed in expositions on “critical epidemiology” that have been circulated widely in Spanish and Portuguese editions [18,38]. The specific lenses applied to ascertain how a policy or intervention in response to underlying conditions is likely to promote health (*Modos de vivir saludables*) are expressed as “4 S’s”: Sustainability, Sovereignty and Solidarity and bio-Security [39], providing in and of itself a more nuanced focus on what is needed to achieve what many in the Global North refer to as “health equity” as an outcome. Assessment of whether mezzo-level modes of living are healthy or unhealthy can accordingly be considered not only by deciphering positive and negative associations linking “health outcome” and determinant indicators – but to directly consider interactions with the 4 S process-related dimensions, again keeping in mind that the relationships are dynamic and not static. This includes consideration of how social actors retain their capacities to resist hegemonic systemic forces (Sovereignty) that may undermine the integrity of their health as well as the capacity to effectively orchestrate a collaborative organic (Solidarity) response in line with their social character. It also recognizes that certain systemic forces that deviate from more organic self-regulating processes can imperil life-supporting systems (Sustainability) – to negatively affect health security (Bio-Security) through resulting imbalances as well as the introduction of threats associated with newly introduced technologies.

In a sweeping review of research conducted on health inequalities in Latin America and the Caribbean, Almeida-Filho and colleagues [40] documented how such research has been extensively conducted in this region, but with a preponderance of the work devoted to conceptual factors and macro-contextual analysis. Less attention has been devoted by social theorists - critical epidemiologists in the South to empirical studies addressing health challenges in specific populations or settings than has been the case in the emerging research in health disparities now developing in North American and European settings [41]. There is thus a challenge to clearly articulate where the alternative orientations lead, in both their processes and outcomes. It is from this perspective that we wish to explore how a SDnH approach can be applied, and to consider the challenges as well as the insights that this can generate. As such, we reflect on our collaborative research program being conducted with the objective of merging the theoretical richness of social determination scholarship with practical empirical approaches acquired through empirical health science scholarship developed more strongly in the Global North.

## Our research program: promoting health equity in diverse settings

Our North-south collaboration began in 2004 as a 6-year capacity-building academic partnership linking the University of British Columbia (UBC) researchers with a consortium of Ecuadorian universities to strengthen institutional capacities for applying a transdisciplinary ecosystem approach to health [29]. From this work together, we built a common vision and foundation for a collaborative program of research, with the goal of pursuing a critical epidemiology approach. In other words, we strove to merge the *theoretical reasoning* emergent in the South with the insights and techniques derived from the *empirical approaches* refined in the North.

The research areas selected were based on the intersection of two factors: first and foremost *problems seen as priorities in the Ecuadorian context* and identified as integrally connected to Ecuador’s accelerated integration into neo-liberal domination [42,43], and/or approaches that were being strongly promoted in Ecuador; and secondly, projects in which the *Canadian researchers had particular interest or areas of expertise* to warrant collaboration. Importantly, this partnership explicitly facilitated the incorporation of more social theory into empirical research projects, both in how the projects were conceptualized and how they were operationalized. The Canadian-Ecuadorian collaborative projects that emerged are described below.

### Health of healthcare workers

Under severe pressures to control public sector spending as a result of terms dictated by international financial institutions, Ecuador’s funding for the health sector plunged to be the lowest in the Americas at the beginning of the new millennium^b^. Associated with this was deterioration in the conditions of human health resources, a neglected health system component that received growing attention with its selection as the theme of the 2006 World Health Report [44]. In Canada, co-author AY had championed a research program focusing on the health of health workers [45-47], so was well positioned to engage in this issue. Following the outbreak of SARS in 2002, this research focused more intensively on bringing together infection control with occupational health [48]. This joint occupational health-infection control team worked together not only in Canada but also in low and middle-income countries (LMICs) [49-51]; and in 2007 worked with colleagues in Ecuador to develop guidance documents and launch capacity building workplace initiatives [52]. A specific area of health concern for health workers globally, and especially in LMICs with a relatively high prevalence and incidence rate of tuberculosis (TB) in the general population, particularly given the increased risk of multiple and extremely drug resistant TB (MDR-TB and XDR-TB) in such settings [53] was indeed the control of infectious disease transmission to healthcare workers. Co-author AY had led the development of new international guidelines for health workers that have been adopted by the WHO, International Labour Organization and UNAIDS [54], and headed a research program in this area (*Promoting Health Equity by Addressing the Needs of Health Workers: A Collaborative, International Research Program*) [49,50,55]. However, while the team recognized the negative impact of global economic forces on health of health workers and public health sector infrastructure [56-58] and built this into their research grant application, until the concerted effort was guided by an understanding of social *determination* of health, inadequate attention was paid in the operationalizing of these various North-south collaborative action research projects to the social processes driving inadequacies in these areas. As the Ecuadorian government sought to strengthen the public health system in response to years of neglect, the opportunity for improving working conditions emerged as an area to be addressed by the Canadian-Ecuadorian collaboration, and a decision was made to explicitly attempt to put the theoretical richness of an SDnH approach of health into practice.

Meanwhile, as President of the Ecuadorian Academy of Medicine, co-author JB was called upon to examine the circumstances of work intensification to which Ecuadorian health professionals are being subjected, introducing a line of enquiry into not just the environmental exposures being experienced, but the patterns of work organization themselves. In the context of this background, our research program is focusing on strengthening occupational health and infection control while building capacity to address the problematic nature of work organization and work relations in this setting. Focusing not merely on identifying risks in the work environment, developing policies and procedures to address these, and training health workers to properly implement these measures, the SDnH approach adopted focuses more directly on contextualizing the work setting and hospital management dynamics within an understanding of global and national economic and political forces. Moreover, the research activity reflects the principles of participatory action research [59,60]. Thus the project revolved around working with health workers to develop their own abilities to identify unhealthy working conditions. The SDnH approach examines individual circumstances (micro domain) but framed within an understanding of the conditions associated with the group to which the participants belong (mezzo domain), and projects these understandings towards promoting changes within institutions and social organizations influencing these domains (macro level). The resulting integrated framework has been guiding us in devising policies and procedures that take into consideration work organization and social hierarchies, directly considering preventive approaches that could be taken up in advocacy for promoting greater health equity. We are currently in the process of implementing research in Ecuador that embraces this latter approach, incorporating the technical expertise acquired by our team in Canada [61,62], South Africa [50,55] and beyond [51], with the extensive existing expertise in Ecuador in occupational health and infection control. Building “agency” through facilitating the creating and strengthening of grassroots networks is a key aspect of the strategy being adopted. Importantly, attention to the 4 S’s has been guiding the conceptualization and research process, as shown in Table 1.

**Table 1:** **Health of health workers study (CIHR funded)**

	**Conceptualization, research and praxis**	**Accomplishments and challenges**
**Bio-Security**	In analyzing options for improving occupational health conditions and decreasing the risk of infectious disease transmission, effects of global economic driving forces on understaffing, underfunding of public hospitals and cost-reducing work patterns are considered, in addition to the needs for implementing control measures for specific organisms.	Trends in increased health expenditures and improved access to healthcare have been identified as linked to political processes - with impacts on working conditions of health workers documented empirically. Health effect associations with organizational, environmental and individual work patterns are being revealed using multi-methods research techniques and placed in a micro-mezzo-macro framework to guide integrated interventions at different levels.
- Health justice		
**Sustainability**	Processes that produce disease, disability or death in health workers, and generate burnout in the healthcare workforce are explicitly recognized as leading to an unsustainable health system.	Empirical research conducted with survey research methods demonstrated burnout in medical staff, associated with their modes of living and the managerial decisions that affect their work life.
- Ecological justice		
**Solidarity**	Focus is on building conditions for empowering health workers and developing networks to share expertise and strengthen capacity to act collectively. Attention is paid to ensuring that interventions concentrate on equity of access to prevention measures as well as services.	Emphasis is on building local knowledge and capacity for action, with attention to needs of all healthcare workers. While yet unclear as to whether the networks being built will be formally recognized by existing institutional structures, hopefully they will be nurtured by camaraderie nonetheless.
- Social justice		
- Agency		
**Sovereignty**	Local ways of producing and disseminating evidence for decision-making are respected, with “outcomes of interest” determined by local values and priorities.	The interventions being implemented were identified and driven by the local Southern partners; information and work surveillance systems developed with Northern expertise will hopefully serve to assist in documenting effectiveness of interventions.
- Epistemological justice		
- Interculturality		
- Respect for local expertise		

### Food and health equity

Ecuador is a country where food production is of critical importance in relation to i) a large agro-industrial export sector associated with ecological impacts; ii) a large number of small producers, particularly in indigenous populations whose way of life has been under threat by changing food distribution patterns; and iii) pressures on healthy eating associated with poverty, transition to processed foods, and introduction of contaminants into the food supply. As a result, this area was identified as a priority domain for our team’s attention, especially in light of the prominent attention to food sovereignty in the 2007–08 process of adopting a new constitution in Ecuador. We therefore linked this focus with food system and health concerns being experienced in Canada to provide an opportunity to explore interconnections in a global food system. To better understand ways to promote health equity and healthy living in response to dominant processes associated with producing, distributing and consuming food globally, we assembled Canadian and Ecuadorian teams of researchers and knowledge users to stimulate thinking about strategies for advancing research on global, national and local scales when faced with complexity, through a five-year research program co-led by co-authors JS and JB (*Food systems and health equity in an era of globalization: Think, Eat and Grow Green Globally [TEG3]*) [63].

We initiated our program by conducting a comprehensive meta-narrative synthesis of published English and Spanish language literature that has confirmed that cross-cultural perspectives can stimulate new insights that may otherwise not be appreciated – and deepen understanding of systemic relationships. While strong proportions of the literature in both languages that cite “food” and “health” explicitly invoke the language of “food security”, identification of “food sovereignty” was revealed by a cursory Google Scholar review as 4-fold greater in Spanish. The different epistemological traditions in fact led to different conceptual frameworks for initiating this exercise in each setting, prompting us to critically reflect on the significance of the conceptual framings we were applying. The English-language review [64] was initiated with reference to identifying distinct pathways (or constellations of determinants) that influence health equity and suggest areas for intervention, while the Spanish language review was organized with reference to relationships to the 4 S’s with more direct focus on processes that affect healthy living. In both orientations, social class influences on nutritious food access were observed to be of particular importance. Sustainability concerns related to climate change and multiple food contaminants (framed as “bio-security” in Spanish) such as agro-toxins (commonly referred to as pesticides in English), animal antibiotic use or genetically-modified organisms (GMOs) were also flagged by knowledge users in both settings as requiring more attention. A further synthesis integrating and contrasting the two epistemological orientations is currently underway, linking researchers and knowledge user partners in Ecuador and Canada.

In the context of this background, specific research projects are now being undertaken. First, we are conducting a comparative economic analysis of conventional versus agro-ecological approaches to agricultural production, taking into account not only the direct economic factors associated with the production units that are typically considered, but incorporating a comprehensive (SDnH-ecosystemic) assessment of indirect costs and benefits that each approach implies for health equity. This entails systematic identification of pathways, estimation of effect and evaluation of impact prompted by consideration of 4 S concerns. Modeling and sensitivity estimations will provide a basis for considering and comparatively analysing results. The study is being simultaneously conducted to examine the leading export commodities in each jurisdiction (bananas in El Oro province, Ecuador, blueberries in British Columbia province, Canada) to consider the insights that a more comprehensive consideration of health equity introduces.

In light of the hegemonic influence being introduced by large-scale food distributors within increasingly globally integrated food systems amid persistent food insecurity driven by income deprivation, the feasibility of alternative methods to provide food is also being explored with particular attention to nutrition and exposure to agrochemical contamination (e.g. of breast milk) in food sold in local markets. A risk-factor-oriented SDH approach typically restricts its focus more narrowly. In contrast, in pursuing an SDnH approach, as shown in Table 2, we are linking access to food to meet the nutritional requirements of marginalized populations with concerns about agrochemical contamination in a direct attempt to build solidarity for actions to achieve sustainable and bio-secure food sovereignty. For example, while pesticide use in the Global South may help reduce the cost of buying bananas by residents of inner city communities in the Global North, this would not be in keeping with the 4S’s in that such achievements would be realized by exposing workers and communities to increased toxic exposures. The SDnH approach thus calls for the building of solidarity between North and South to recognize the lack of sustainability of this approach, questioning implications of capital accumulation by large multinational food corporations.

**Table 2:** **Food systems & health Equity/healthy living (CIHR funded)**

	**Conceptualization, research and praxis**	**Accomplishments and challenges**
**Bio-security**	Multi-dimensional analysis of processes associated with the production, distribution and eating of food to monitor direct as well as indirect (e.g. including environmental and socio-cultural interaction) pathways at different scales (global, national, local) to consider concerns such as effects on healthy eating and food security, as well as the introduction of contaminants.	Comprehensive English and Spanish language literature reviews have been carried out to consider opportunities for interventions to address gaps – and take account of different emphases in different settings, including the scope of what is meant by “health equity” in different cultures, leading us to extend the research program vision to embrace “healthy living” and the 4S orientation. As part of this effort, we are exploring the feasibility of examining multiple agricultural contamination of food and contamination of breast milk in women as a result of intensive chemical contamination agriculture.
- Health justice		
**Sustainability**	Emphasis on considering food system effects on the sustainability of ecological and living systems that are otherwise undermined by the failure to take account of negative effects and positive opportunities associated with food production, distribution and consumption systems.	A comparative analysis of the positive and negative effects of agro-ecological and conventional production systems is underway to consider policy options to promote health equity by ensuring that such factors are considered in food-related decision-making. There are extensive measurement challenges in doing this.
- Ecological justice		
**Solidarity**	Attention to social capacities for building healthy production systems and relationships to counter pressures from concentrated interests that dominate the global food system.	We have been examining the efficacy and effectiveness of strategic alliances and networks to support alternatives to global pressures identified as promoting negative health impacts – and attempting to confirm interest of policy-makers in the findings.
- Social justice		
- Agency		
**Sovereignty**	Particular emphasis is on implications of operationalizing Food Sovereignty (recognized in the Ecuadorian Constitution) for promoting health.	Local capacities, resilience of social forces and the strength of the local agro ecologic culture to resist imposed food system transformations of food system relationships and assert healthier patterns is being reviewed, including consideration of policy options to enable this.
- Epistemological justice		
- Interculturality		
- Respect for local expertise		

### Antibiotic resistance

With the underfunding of health care generally and neoliberalism’s related discouragement of a comprehensive primary health care system, there has been a strong tendency for self-medication by marginalized populations who are suffering a burden of disease associated with their conditions of poverty (marginalization having contributed to very high poverty rates in Ecuador over the course of the 1990s and early 2000’s) [43,65]. This led to widespread misuse of antibiotics that are contributing to the emergence of antimicrobial resistance, fueled by poverty and poor living conditions [66,67] aggravated by widespread agro-industrial non therapeutic application of antibiotics in animal husbandry [68-70]. Having initiated work on this challenge [71] in the course of the collaborative training program that we conducted, our team received funding for piloting community-based research to address these issues (*An Ecosystem Approach to Antimicrobial Stewardship: An Ecuadorian- Canadian Collaboration to Design, Implement and Evaluate a Community-based Intervention*).

Working with indigenous communities, we quickly learned that an Indigenous “cosmovision” or worldview of what constitutes adequate evidence differs considerably from a Western traditional epidemiological approach that places highest value on quantitative evidence of illness-related indicators. From this traditional epidemiological perspective, we tend to believe that the “gold standard” for measuring success in the area of antibiotic resistance is either reduced morbidity/mortality related to drug-resistant bacterial infections, or detection of less resistance and fewer resistant strains in routine microbiological testing, or reduced use of 2nd and 3rd line antibiotics. What we found was that not only were the surveillance systems not in place to collect the needed data to evaluate the effectiveness of our interventions, but that these measures fail to capture concepts such as good living or Sumak Kawsay (in Kichwa), that come from the wisdom of an Indigenous worldview. Within the latter epistemology, success is defined by perceived healthy and sustainable relationships between living organisms, with a focus on process, mutual respect, and reverence for the autonomy of others – processes that address the root of the structural problem, locally, nationally and internationally.

As part of our research collaboration, we did manage to work with indigenous communities to address concerns about water quality [72]. As well, several knowledge, attitude and practice studies in specific populations in Ecuador were also completed and, working closely with indigenous colleagues, we developed an intercultural community booklet on appropriate antibiotic use [73] that was field tested with two separate groups of Community Health Workers and revised accordingly. We collected data on antibiotic use from 2008–2011, and worked with communities to develop some possible interventions at the level of the pharmacy dispensary, physician practices, families, community health promoters, and government programs; we strengthened relationship with experts on antibiotic use in animals; and we collaborated with another organization in Ecuador, ReAct, promoting their arts-based methods to improving antibiotic stewardship [74]. Other researchers – both in the Global North and Global South, of course, have also approached the issue of antibiotic resistance with attention to underlying drivers. As noted above, Cole and Wing, for example, documented the impact of concentrated animal feeding operations on antimicrobial resistance [68]. We therefore linked antimicrobial resistance, and the health hazard this presents to humans, to the underlying manner in which food is now being produced, in addition to well-documented profit-seeking behaviour of pharmaceutical companies [75].

A traditional SDH approach emphasizes education on hand hygiene, sanitation, clean water, and infection control measures such as cough etiquette, as well as knowledge on the difference between viruses and bacteria, as some of our team members had done in Canada, operating under a different paradigm [76]. In contrast, the SDnH approach we decided to adopt in our Ecuador work, while providing the necessary information found in the “Do bugs need drugs?” Canadian manual, also explicitly focuses on raising awareness of the reasons for the emergence (or social production) of antimicrobial resistance, and embraces an intercultural approach to health – promoting living in harmony with nature and all the organisms of the ecosystem, as well as encouraging community mobilization to demand that basic needs are met. As such, the intercultural booklet embracing an ecosystem approach to antimicrobial resistance that we produced working collaboratively with indigenous communities, government departments, the Pan American Health Organization, and university partners in Ecuador, while providing practical advice, does not shirk from identifying the social processes that perpetuate the transmission of infectious diseases and the development of antimicrobial resistance. Although we had intended to conduct a rigorous evaluation of the use of the booklet by Community Health Promoters within a cluster-randomized trial in communities across Ecuador, the contextual reality precluded proper implementation. Some of the challenges related to changes in personnel within the Ministry of Health, as well as the realization that the surveillance systems did not exist for capturing data on antimicrobial use, let alone on trends in resistance, leaving us with only process measures (as well as knowledge, attitudes and self-reported practice) to serve as outcome to measure quantitatively. We therefore set out to build the infrastructural capacity, while promoting the use of the material, leaving rigorous evaluation to a future date. Traditional epidemiology would consider this study a failure; however, the research process itself brought together communities, non-governmental organizations, national government and international agencies, serving to raise the level of awareness and conferring some empowerment to serve as a basis for future challenges to detrimental social processes. Table 3 assessed this collaborative research project with respect to the 4 S framework.

**Table 3:** **An ecosystem approach to anti-microbial resistance (CIHR funded)**

	**Conceptualization, research and praxis**	**Accomplishments and challenges**
**Bio-security**	Antibiotic resistance is now rampant due to many processes: short-term profit-seeking behaviour on the part of Big Pharma and food production industries; inadequacies in healthcare provision which lead to self-medication due to inaccessible medical attention or needed medication; and especially the social disparities related to infectious diseases and their transmission due to inadequate sanitation, clean water, proper housing and nutrition.	We collaboratively developed an educational guidebook for community health promoters that not only provided them with information on differences between viruses and bacteria, when antibiotics are not needed and how to manage common upper respiratory tract infection or gastrointestinal disease likely of viral origins, but also addressed the social drivers of antimicrobial resistance. Unfortunately, our research fell short of designing, implementing and rigorously evaluating the impact of using our educational tools in interventions in communities. This was partly because of the absence of surveillance systems for antimicrobial use, let alone antimicrobial resistance – precluding objective evidence of impact, and partly because changes in personnel at the Health Ministry hindered the implementation of a well-designed intervention study.
- Health justice		
**Sustainability**	All organisms have a role in the complex ecosystems of our planet, and all life should be respected. The Ecuadorian constitution provides protection to nature independently of property rights.	Our discourse emphasizes the important role of microbes in the universe. Our collaborative Ecuadorian-Canadian team has been working to promote a “re-imaging of resistance” raising awareness that destruction of ecological integrity constitutes a threat to human health. We would have liked to contribute rigorous empirical evidence linking animal husbandry practices to increased antimicrobial resistance but were unable to do so.
- Ecological justice		
**Solidarity**	Combatting antimicrobial resistance, embracing a social determination of health approach, requires promoting grassroots mobilization to demand changes to the drivers of antimicrobial resistance, including not only changes in policies regarding drug use, but also equal access to clean water, safe food and healthcare services.	We conducted several workshops with community health promoters as well as provided a certificate program for health professionals that required their conducting projects in their communities, thereby building local capacity to address the immediate as well as more structural determination of antimicrobial resistance. However, we have not been able to rigorously evaluate our efforts to date.
- Social justice		
- Agency		
**Sovereignty**	Respect for indigenous beliefs and values is essential in promoting wellness and combatting the symptoms of minor infections. Ancestral knowledge, including the appropriate use of medicinal plants is to be encouraged.	We learned that merely incorporating information on how to use medicinal plants was a superficial way of trying to respect indigenous concepts of wellbeing, yet in order to maintain institutional support from experts who maintained that ancestral knowledge lacked an evidence-base, we could not give full appreciation to indigenous cosmology.
- Epistemological justice		
- Interculturality		
- Respect for local expertise		

### Vector borne disease (dengue)

Amid conditions of social-environmental degradation, including deteriorated and undeveloped urban infrastructure, dengue fever has grown to be a major health concern across Latin America and the Caribbean as part of a global pandemic, aggravated by climate change and the agri-business monoculture-induced low biodiversity settings, making previously unaffected areas vulnerable. In Ecuador, effects have been especially felt in coastal areas marked by pronounced irregular and discriminating urban and peri-urban expansion. Building again from work initiated under our academic training program, we have been conducting research (*Meeting capacity-building and scaling-up challenges to sustainably prevent and control dengue in Machala, Ecuador*) on the application of an eco-bio-social approach to prevent and control the disease as part of a network of similar projects funded by the WHO’s Tropical Disease Research Program. This program especially focuses on community participation and intersectorality to address an issue where no effective vaccine or treatment exists. However, despite recognition that social factors are critical to understanding and addressing the disease, the analysis of social factors at play has remained shallow.

To improve the methodological basis for appreciating social considerations, in conducting the Phase 1 Situation Analysis for this project, we applied the Social Insertion Index (INSOC) and Housing Quality Index (HQI) developed by co-author JB as an expression of SDnH by coding responses from a randomized survey of 2000 families in 20 Machala clusters. Analysis comparing the application of these indices with observations using the impressionistic social class designations of other studies revealed a significantly greater validity of INSOC in providing an evidence-based means for examining the social ecology (stratified as “high”, “medium” and “low” [77]). For example, distinct social gradient relationships by INSOC social class designations (in contrast to impressionistic categorization) were observed with regard to housing quality and the type of water containers at greatest risk for dengue infestation. We are now proceeding to extend this analysis more broadly to 2011 Ecuador census data which provides sufficient data to construct INSOC variables. Using this, we will then delineate the kinds of interventions that can benefit from this more sensitive approach to social characterization, noting the limits that behaviour adaptation interventions can offer in comparison to the pursuit of broader structural and infrastructural transformations.

The vocabulary of “risk factors” used in public health and conventional epidemiology to consider points of intervention, creates a bias towards targeting more proximal behaviour modification options that can affect exposure to patterns of risk, rather than looking more critically at the processes contributing to vulnerability. In applying an SDnH approach, we are examining a wider range of pathways to health equity. In the context of this background, we are focusing on working with communities to deepen understanding of the processes of social determination of vector-borne diseases that have been lacking to date, including implications of alternate land use and infrastructure [78]. Efforts therefore focus on applying a transdisciplinary approach to achieving sustained, bio-secure (without pesticide use), healthy communities (with healthy housing and water supplies) to prevent the spread of disease. The 4 S analysis of the accomplishments and challenges we have been facing in this project are summarized in Table 4.

**Table 4:** **Control and prevention of dengue (TDR/IDRC funded)**

	**Conceptualization, research and praxis**	**Accomplishments and challenges**
**Bio-security**	We are examining the presence/reproduction of unhealthy modes of living and unhealthy agricultural spaces in the context of an unhealthy metabolism between unsafe agricultural production and its ecological conditions. Emphasis is on understanding how social processes affect vector transmission in the socio-ecological context of an agro-industrial region as well as the associated exposure and vulnerability of marginalized populations amid an increasing and uneven prevalence of dengue. We anticipate that with increased attention to the engagement of the affected communities’ there will be a direct involvement in processes to decrease their vulnerabilities and to monitor negative impacts.	We have successfully demonstrated the INSOC (Social Insertion) index as a more sensitive measurement tool for analyzing social gradients of vulnerability. As well, a computerized system (SAT-Dengue) for rapid notification to increase the efficiency and effectiveness of health system performance has been introduced – with the intention of building this as part of an intersectoral integrated system for monitoring additional elements to be addressed in control and prevention actions. The effectiveness, albeit limited, of community prevention and control activities has been documented, setting the stage for a more comprehensive analysis of options for intervention that can provide stronger prospects for reducing exposure to dengue.
- Health justice		
**Sustainability**	Consideration of broader processes of land use, chemical application and food production is being considered in relation to the sustainability of conditions for healthy modes and living contexts.	Recognizing the limitations of what can be achieved through adaptive local community actions, attention to broader contextual processes has been introduced in reviewing study results, including opportunities for strengthening environmental regulations, municipal infrastructure and its economic feasibility and the interactions with broader patterns of agro-industrial development that have transformed the local ecology.
- Ecological justice		
**Solidarity**	Emphasis in the study has been on the role of community engagement (through the participation of health promoters and neighbourhood school activities) in achieving prevention and control of dengue by restricting conditions for the virus-carrying vector to multiply	Patterns of networks that are involved in dengue prevention and control have been analyzed to highlight need and opportunities for sustainably building effective community engagement, countering vertical paternalistic approaches such as that which was introduced by a “top-down” bio-larviciding program that was concurrently initiated by the Ministry of Health during the study period.
- Social justice		
- Agency		
**Sovereignty**	Effects on local communities of interactions with government and other institutions including has been carefully monitored, through direct involvement of local community organizations.	Greater accountability for further dengue activity by authorities is being emphasized; as are opportunities for communities to build on their increased involvement in this study to take on additional health priorities that they have identified in their health committees.
- Epistemological justice		
- Interculturality		
- Respect for local expertise		

### Social circus

Recognizing the alienation and circumstances of oppression that can be systematically reinforced in a polarized class society marked by increased commodification of social experience, we have initiated research into the potential of “social circus” as a process to engage youth and improve their health as well as that of their communities, and potentially engage in social transformation. Building on the earlier work of performance studies theorist Jennifer Spiegel [79,80], a 3-year grant from CIHR was obtained to apply mixed research methods to improve understanding of the micro (individual), mezzo (community) and macro (social system) impacts of the extensive social circus programs now underway in marginalized communities in Ecuador.

Social circus projects have been rapidly expanding over the last 15 years, with social circus proponents claiming an array of health benefits, noting that engaging in circus arts help people express their creativity while demanding perseverance and discipline that can have beneficial effects on their own mental and physical health, and on the well-bring of their communities [81-84]. Social circus can be seen as part of the increasing interest in art-for-social-change, wherein arts-based practices have indeed been transformative, as noted by artists and educators like Brecht [85], Freire [86] and Boal [87]. Canada has cutting-edge practitioners in the use of performing arts, such as theatre and dance to address social determinants [88] and promoting community health [88]. As Fraser and al Sayah concluded, however, rarely do studies identify theoretical underpinnings of the research [89] and even more rarely do studies take a critical perspective to confront issues of power.

The question of exactly *how* arts can contribute to global health equity [90] has increasingly been posed. Ecuador is a country with a rich tradition of art-for-social change. This question began to be posed by our team when *Cirque du Soleil* began in 2011 to implement a government-sponsored social circus program to promote healthy social policy. Ecuador’s Vice President, with a particular interest in humour and the arts, made this one of his flagship initiatives in a national public program, reaching many thousands of participants. The program focuses on street youth, but also includes programs for children from marginalized populations. We therefore decided to explore how social circus can influence the social determination of health. In addition to participant observer methods, focus groups, interviews and questionnaire surveys, we are incorporating participatory arts-based methods (such as photovoice [91]) to engage the participants themselves in the research process not as subjects of the research but as active members of the research team. We are considering if roles are stereotyped and participation mitigated by sex and gender, for example; we will be considering the power differentials in social circus programs; and we will be developing a much-needed theoretical approach to understanding *how* the use of circus arts by marginalized populations can challenge the social processes perpetuating their marginalization [79], to the extent they chose to do so [92].

A more traditional SDH approach which we otherwise might have engaged in, would have had us focusing on the social workers’ follow-up of social problems identified in participants of social circus programs (domestic abuse, poor housing conditions, educational barriers, inadequate nutrition, etc.) whereas a SDnH approach is very much about empowerment of social circus participants to address social inequities. Some programs in Latin America do have social transformation as an explicit goal [93,94]. While this goal is more muted in this government-funded program, the aim of the research is to improve understanding of how impacts at the micro, mezzo and macro levels interact and how social circus can contribute to transformative change. Table 5, applying the 4 S framework, highlights not only the conceptualization of the micro-mezzo-macro determination of health, and the importance of participation, but also a sincere respect for the contribution and perspectives of those living “at the margins”.

**Table 5:** **Social circus and health equity (CIHR funded)**

	**Conceptualization, research and praxis**	**Accomplishments and challenges**
**Bio-Security**	Economic and social marginalization leads to alienated youth with low self-esteem and poor mental and physical health as well as counterproductive styles of living. Social circus promotes personal growth (micro) through engaging creativity and building perseverance to take on challenges as well as generating a strong sense of solidarity (and possibly national pride) through team building, social engagement and social inclusion (mezzo level changes). These attitudes and skills lead to stronger communities better able to address the social processes that drive marginalization (macro level).	Using mixed methods research, including quantitative surveys, in additional to qualitative methods, we are clearly able to document and interrelate the micro and mezzo level impacts of social circus on participants and to a certain extent, their immediate community. Impact on social processes that drive marginalization will have to be assessed in a longer-term endeavour – as our longitudinal time frame is only 3 years.
- Health justice		
**Sustainability**	Many of the local programs are under threat, as they depend on public funding and awareness by various levels of government of the value of community programs such as this, despite what appears to be very strong benefits of social circus to those who participate.	We have yet to determine how best to study the “value” of these programs, in terms that will lead decision-makers to make solid commitments to sustain the programs.
- Ecological justice		
**Solidarity**	The innovative social intervention (social circus) can challenge hierarchical relationships, and indeed generate improved social democracy. Although the intervention per se (social circus) is the object of study and not under the control of the research team, the research team is endeavouring to conduct the research in a manner that itself is empowering.	The research has not only embraced participant observation, performance ethnography and other qualitative research methods but has placed particular emphasis on participatory arts-based research methods (e.g. “photovoice” and circo-theatrical research creation by participants) to further build local capacity and agency. Social class variables (INSOC) have been included in the longitudinal cohort study of social circus participants, to ascertain the relationship between social class, and the benefits of social circus.
- Social justice		
- Agency		
**Sovereignty**	The theoretical conceptualization of social circus is to value the contributions “from the margins” rather than merely to attempt to build skills in participants that will lead them to better conform to the market economy. Nonetheless different “promoters” of social circus have different objectives, with decreasing street-based lifestyles and building national pride figuring prominently.	While nurturing North-south bonds in this rapidly growing global social circus community, care is being taken to minimize cultural imperialism (particularly relevant because of the strong influence of Cirque du Soleil), and the research process is endeavouring to respect local art related practices and the pathways to fulfilment chosen by participants and their communities rather than impose external values.
- Epistemological justice		
- Interculturality		
- Respect for local expertise		

## Critical processes of social determination of health

There are three common themes that characterize the seemingly diverse research initiatives that are presented above. First is the persistence of hegemonic neoliberal socio-political pressures that have driven disparity in Ecuador by promoting patterns of accumulation that disadvantage marginalized populations and undermine their resilience. Secondly, these same forces have stimulated the development of counter-hegemonic responses in all cases, and this is explicitly recognized and incorporated in the research. Thirdly, the focus of each of these research initiatives is not merely on addressing the proximal processes threatening health - i.e. the specific SDH in question (e.g. poor infection control in hospitals, lack of nutrition, misuse of antibiotics, vector-borne diseases, or alienation of marginalized youth), but rather the social processes that could be challenged in a manner that would empower communities (including workforces) to address underlying power relations. In this way, the SDnH approach we are endeavouring to apply in our work aims at the processes that drive social inequities, not merely the effects of these inequities, while at the same time directly considering the resistant (liberatory) forces capable of countering negative health impacts. Specifically:

i) facilitating the empowerment of health workers to respond to deteriorating working conditions and to address the power differentials and work organization within healthcare as well as the detrimental state decision-making processes;
ii) working with NGOs and producer groups to improve food equity, biosecurity in agro-ecological production, to mobilize in response to commodification trends and narrowing options driven by a neo-liberal food system;
iii) strengthening the knowledge and skills within indigenous communities and NGOs to counter the development of anti-microbial resistance in response to inappropriate use of antibiotics driven by short-term profit-seeking behaviours, and to demand that basic needs are met;
iv) working with a variety of partners in marginalized urban and peri-urban community settings to more effective and sustainable responses to threat of vector-borne disease amid conditions of socio environmental deterioration, entomological disruption and expansion of unhealthy health living patterns and exposure; and
v) working with street youth and other marginalized groups who participate in social circus in order to facilitate building self-esteem, skills and social networks to strengthen their ability to strive for transformative change.

Furthering the themes developed in the Latin American “collective health” school that stress the importance of collective determination over free will and individual life styles [8], our work thus attempts to explicitly focus on inequitable power relations. In line with such orientations, scholarship such as that of Cesar Victora [95,96] has demonstrated the feasibility of linking socially determined inequity to the understanding of its empirical evidence (inequality), with the powerful tool of refined mathematical analysis. In our work together, we build on this strong foundation to try to incorporate intervention research techniques acquired in the North through several decades of community-university partnered multi-method participatory research.

As explained by Breilh [78] “criteria for truth” in the social determination model is the extent to which the research process can effect real social change. Indeed in many Northern schools of population health (including the home base of authors AY and JS), advanced skills in statistical techniques are required for a doctoral degree by all students regardless of their topic or preferred methods of research, while no such emphasis is placed on critical theory. Certainly medical students in the faculty in which AY and JS work are often completely unfamiliar with the social processes underlying poverty [56], while they learn that randomized controlled trials with sophisticated statistical techniques are the required standard of evidence for interventions to be deemed effective. Emphasis on such relatively narrow standards of proof has prompted Schrecker [97] to provocatively query “Can health equity survive epidemiology?” – and conclude that it is essential to consider and pursue broader approaches (methodological pluralism). In contrast, at the home base of Southern author JB, familiarity with emancipatory theory is a core requirement for doctoral studies in the collective health program, while statistical skills are only required of those who intend to use such techniques in their theses. Our collaborative research is attempting to apply both advanced epidemiological techniques as well as social theory. Our point, however, is that the language of social determinants (especially in its simplistic form, with limited differentiation made between immediate and structural determinants) lends itself to research that is more reductionist and hence beckons the development of different skills than would be applied by those who adopt the language of social determination.

## Summary

Despite increased attention to social determinants of health and health equity in recent years, it has been observed that disparities have in fact been deepening. Embrett and Randall [98] suggest that a reason for this can be found in the failure to adequately integrate and apply a thorough understanding of policy analysis theory. While we strongly agree that attention to policy theory can contribute to more effective interventions, we argue that there is a more fundamental need to strengthen our grasp of the processes that drive health inequities and capacities for addressing them. Our experience illustrates that we do indeed need a sharper theoretical focus in guiding empirically-rich intervention research, but this theory must look not at discrete social determinants of health, but rather to comprehend and then address the critical processes of social determination that systemically drive disparities. This sharper focus is needed in order to promote the conceptualization and operationalizing of intervention research in a manner that could be more transformative.

As noted by Barrios Suarez and colleagues [28], the purpose of global health research is contested, with some researchers arguing that global health objectives should be “ideologically neutral”, while others, exemplified by Lavery et al. [99], propose a “relief of oppression” framework that recognizes that Northern researchers have a role to play in promoting social justice as these inequities are at least in part shaped by inequitable trade policies and a history of colonization driven by Northern high-income countries. These themes have been in the forefront of discussions by some critical scholars in the North, but as noted by Krieger [3] has been particularly prominent in Latin America, where debate continues to flourish [24].

Consistent with the social justice perspective, and building on a sound institutional and academic platform that bridges Northern and Southern traditions, we argue for language that promotes a scientifically sound yet more emancipatory approach to health issues [18,100]. In doing so, the knowledge of people and their ancestral and present wisdom, is much more than a resource for better ethno-medical and therapeutic knowledge within a biomedical paradigm. Traditional epidemiology has also much to learn from them, about integral notions of space, sustainable relations between nature and humanity, a healthy conception of time, a harmonious management of the planet’s energies and about a fair, equitable and protective construction of social relations. Therefore, it is not surprising, to observe the proximity in meanings of the indigenous Kichwa word 'Sumak Kawsay' (good living) which has been established as a central concept within the new Ecuadorian constitution adopted through popular processes in 2008 with our academic conceptions of ‘healthy mode of life’.

In providing a perspective on the development of Latin American critical (‘social’) epidemiology in the *International Journal of Epidemiology*, Breilh [17] called for “an opportunity to form fraternal partnerships on the intercultural road to a better world, where only an epidemiology of dignity and happiness will make sense”, in response to “the menacing forces producing our unhealthy societies”. The failure to have adequately embraced insights emerging from all corners of the globe undermines the benefits that this can bring to world knowledge - and risks contributing to “epistemicides” that marginalize alternative forms of knowledge [101]. Prominent Brazilian epidemiologist Cesar Victora even conjectured that strategies to break the under-representation of such contributions from the “South” are themselves needed [102], in line with Raewyn Connell’s observation that with few exceptions, “mainstream social theory sees and speaks from the global North” [103]. Notwithstanding the difficulties we encountered in operationalizing research in line with our social theory, we argue that there is much to gain from pursuing collaborations that concurrently promote the strengths of design and methodology that have been spawned by conventional epidemiology from the North with the epistemological and theoretical insights developed particularly from the Latin American collective health theorists.

To return to the central question of this article – “does language matter?” – we contend that despite the recognition in the dominant Northern paradigm of “health equity/social determinants of health” of the social processes that drive the unfair systematic disparities in health, the language of researchers and practitioners applying this paradigm can still lead to focusing on the social determinants themselves rather than on the social determination process. We believe that shifting the language to explicitly focus on “social determination” rather than “social determinants” will help keep the focus where it belongs to better promote pathways to health for all.

## Endnotes

^a^The language of metabolism, interestingly, encourages conceptualization of integrating processes within an organism, such as the socio-ecological relationships that discussions of sustainability encourage, but is a framing more readily applied in Spanish than in English.

^b^In 2000, Ecuador’s Total Health Expenditure as a % of GDP was 3.61%, versus an average of 6.62% for all countries in the Latin America and Caribbean region. World Bank, World Bank, Data, Health Expenditure, total (% of GDP) http://data.worldbank.org/indicator/SH.XPD.TOTL.ZS?page=2.
